# More Than Meets the Eye: Unraveling the Interactions Between Skin Microbiota and Habitat in an Opportunistic Amphibian

**DOI:** 10.1007/s00248-025-02489-1

**Published:** 2025-01-24

**Authors:** Zanovello Lucia, Galla Giulio, Girardi Matteo, Casari Stefano, Lo Presti Irene, Pedrini Paolo, Bertorelle Giorgio, Heidi C. Hauffe

**Affiliations:** 1 Conservation Genomics Research Unit and Animal, Environmental and Antique DNA Platform, Research and Innovation Centre, Fondazione Edmund Mach, San Michele All’Adige, TN Italy; 2 Conservation Biology Unit, MUSE–Science Museum of Trento, Trento, Italy; 3 Population Genetics and Genomics Group, Department of Life Sciences and Biotechnology, University of Ferrara, Ferrara, Italy; 4 National Biodiversity Future Center (NBFC), Palermo, Italy

**Keywords:** Amphibian, Microbiota, Mycobiota, Fungi, *Bombina*, *Batrachochytrium dendrobatidis*

## Abstract

With amphibians still holding the record as the most threatened class of terrestrial vertebrates, their skin microbiota has been shown to play a relevant role in their survival in a fast-changing world. Yet little is known about how abiotic factors associated with different aquatic habitats impact these skin microorganisms. Here we chose the yellow-bellied toad (*Bombina variegata*), a small anuran that colonizes a wide range of wetland habitats, to investigate how the diversity and composition of both its bacterial and fungal skin communities vary across different habitats and with water characteristics (temperature, pH, and dissolved oxygen) of these habitats. Skin microbiota was sampled from 14 sites in the Province of Trento (Italy), including natural pools, ephemeral ponds, irrigation tanks, and farm ponds. Interestingly, the diversity of the two microbial components was also highly correlated. Close associations between both the diversity and composition of water and skin communities were noted for each habitat and sampling site, suggesting that water bodies actively contribute to the skin microbiota assemblage. In addition, water pH, temperature, and dissolved oxygen affected both bacterial and fungal diversity of skin. We confirmed the presence of *Batrachochytrium dendrobatidis* in skin samples of animals collected from eight waterbodies, as well as more than 60 microbial taxa previously associated with resistance to this pathogen. We concluded that both skin bacterial and fungal communities appear to be influenced by each other as well as by environmental communities and conditions, and these relationships connecting the whole ecosystem should be considered in future research concerning amphibian conservation.

**Supplementary Information:**

The online version contains supplementary material available at 10.1007/s00248-025-02489-1.

## Introduction

As amphibian populations continue to decline worldwide, mainly due to human-driven habitat modifications and emerging diseases [1], skin microbiota is of particular interest as both a possible means of adaptation to the rapidly changing environment and a defense against pathogens [2–4]. A substantial body of amphibian skin microbiota research has been generated in the last 10 years, ranging from the characterization of bacterial taxa [e.g., 5] to the assessment of microbiota variation between species and life stages [6, 7] or through time [8, 9]. As several threatened species are involved in ex situ captive-breeding programs, the impact of captive rearing on skin microbiota has also been summarized in a recent review [10]. Unsurprisingly, several studies have focused on the interaction between amphibian skin microbiota and known pathogens, especially the deadly chytrid fungus *Batrachochytrium dendrobatidis* (Longcore, Pessier & Nichols, 1999; hereafter, *Bd*). This pathogen has led to population declines and local extinctions of several amphibian species and is considered one of the major threats to amphibian conservation [11]. Such studies have shown how bacterial skin communities can influence the resistance of wild amphibian individuals to *Bd* infection [e.g., 12] and conversely, the impact of fungal infection on skin bacterial composition and diversity [13]. Several skin-associated bacteria are known to produce antifungal metabolites, effectively preventing *Bd* infections [14]. Interestingly, the possibility of translocating these taxa from one amphibian species to another has recently been tested as a means of increasing amphibian resistance to *Bd* [15]. However, only very recently has amphibian microbiota research started characterizing fungal skin communities (mycobiota) and their potential role in disease resistance [16–18]. In addition, the complex interplay of both bacterial and fungal communities is still largely unexplored [19, 20].

Amphibian skin bacterial communities are known to be influenced by several factors, including the composition of the environmental microorganism communities, which constitute an important source of bacteria and fungi for maintaining skin communities [21, 22], and habitat quality [23]. On a global scale, Kueneman and colleagues [24] have shown that amphibian microbiota changes in relation to macrohabitat, annual climatic trends such as temperatures and precipitation, and elevation. However, at a finer scale, the relative importance of specific abiotic variables like water temperature and pH to the observed variations in skin microbiota composition has only very recently begun to be addressed, and never for mycobiota [3 and references therein; [25].

Given the above gaps in knowledge, this study aimed to (i) characterize the skin mycobiota of a widespread amphibian and investigate, in four freshwater habitat subtypes, the associations between (ii) bacterial and fungal components of skin, (iii) skin microbiota and water microbiota, and (iv) skin microbiota and abiotic variables, as well the incidence of *Bd*. We chose to focus on *Bombina variegata* (Linneaus, 1758) due to its ability to colonize a wide range of natural and constructed wetland habitats, although preferred reproductive sites are exposed temporary pools at the edge of woodlands [26]. Despite being widely distributed across central and southern Europe and classified as Least Concern (LC) in the IUCN Red List [27], yellow-bellied toad populations are locally in decline. Such is the case in the chosen study site, the mountainous Province of Trento, Italy [28], probably due to habitat loss because of urbanization and wetland pollution [29]. Hence, the species is listed as Vulnerable (VU) in the Provincial Red List [28]. The skin microbiota (including mycobiota) has never been studied previously, but infections of *Bd* on this species have been reported over much of its distribution [e.g., 30, 31], although never for Italian populations [32], nor have mass mortality events nor evident signs of disease ever been observed in the study area [33].

## Materials and Methods

### Skin Swabs, Water Samples, and Parameter Collection

**Fig. 1:**
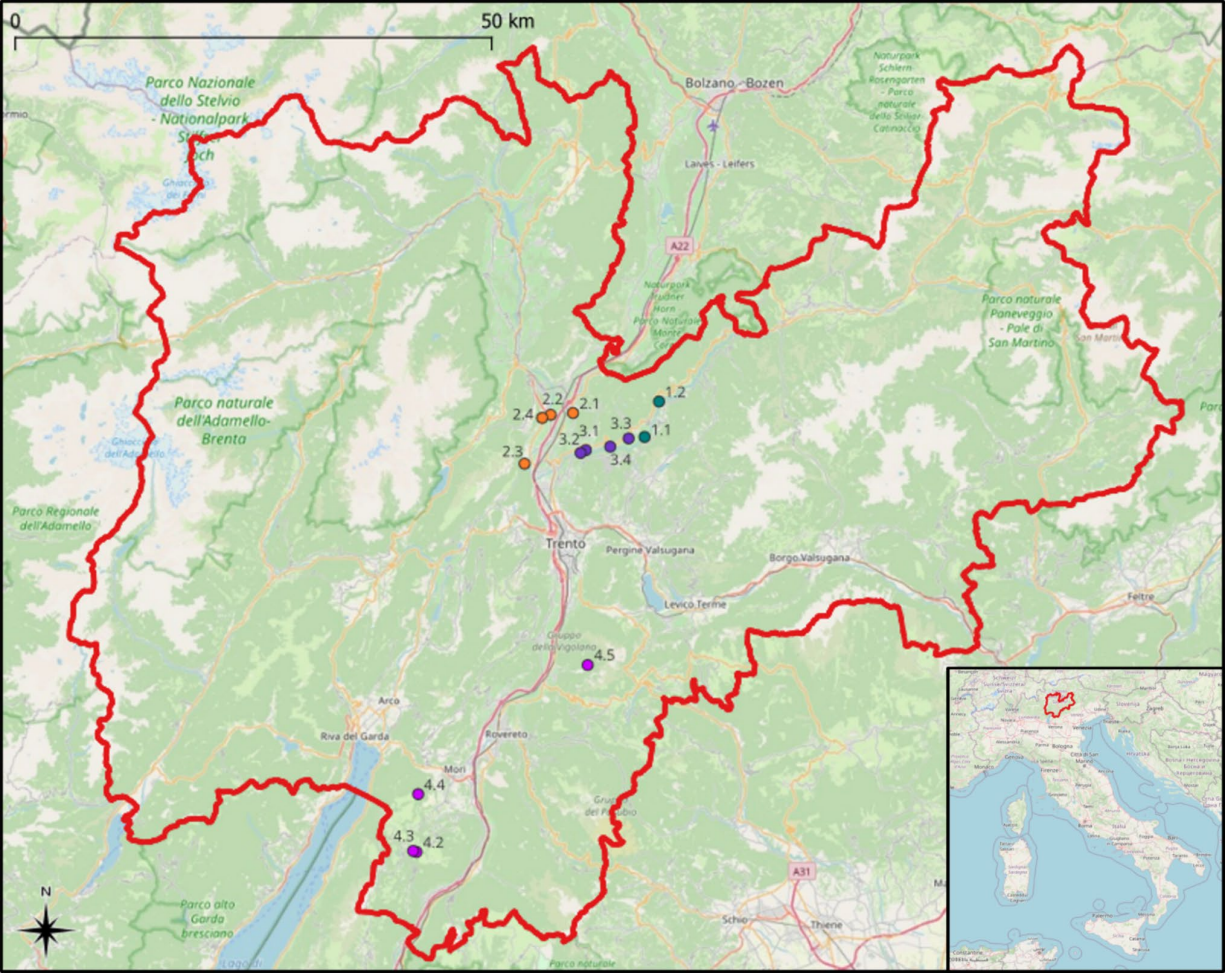
Map of the study area (Province of Trento) with its location on the Italian peninsula (lower right panel). Sample sites are represented by closed circles, colored according to habitat type (FLU: dark green, VBE: orange, AGR: purple, PAS: pink) and labeled according to site code numbers (see Table 1). Scale bar and compass are also shown on the left. This image was created with QGIS.org (2024) QGIS Geographic Information System. Open Source Geospatial Foundation Project http://qgis.org

Animal handling procedures for research purposes were authorized by the Italian Ministry of the Environment according to DPR 357/97 on April 7th, 2021 (Prot. ISPRA 16759 of 6/4/21; Authorization no. 0035657 of 7/4/21 by the Ministry of the Environment). We sampled *B. variegata* individuals from 14 sites in the Province of Trento, including five of the nine known reproductive habitat subtypes of this species described for this area by Endrizzi et al. [28]. These freshwater habitat subtypes were chosen to represent the ecological diversity of the species while also taking into account the known abundance, as some sites support very small and elusive populations (cfr. Figure 13 in [28]). Therefore, sampled sites included two “fluvial habitat subtypes” (FLU), four “valley bottom ecotone habitat subtypes” (VBE), four “agricultural habitat subtypes” (AGR) and four “pasture habitat subtypes” (PAS). General descriptions of these sites can be found in Table 1, as well as their respective location, number of samples collected, and water parameters. The geographical distribution of the sampled sites is represented in Fig. 1.

**Table 1:** General description of the sites where *Bombina variegata* individuals were collected, with the habitat subtype and water parameters (altitude and water pH, dissolved oxygen, and temperature) as well as number of samples (individuals and water) collected per site

Habitat code^a^	Site ID^b^	Location	No. of skin samples^c^	No. of water samples^d^	Altitude a.s.l. (m)	Water pH	Dissolved oxygen (mg/L)	Water temp. (°C)
FLU	AMB-1_1	Pozzolago, Lona Lases	10	2	411	7.76	7.86	18.6
FLU	AMB-1_2	Falesia di Prà, Segonzano	9	2	499	8.73	8.73	20.9
VBE	AMB-2_1	San Michele all’Adige	10	2	210	7.52	0.44	13.2
VBE	AMB-2_2	Maso delle Part- Maso Inon Mezzolombardo	15	2	211	7.15	5.78	18.1
VBE	AMB-2_3	Zambana	6	2	199	8.29	7.88	31.1
VBE	AMB-2_4	Mezzolombardo	10	2	209	7.23	1.94	19.8
AGR	AMB-3_1	Verla 1, Giovo	10	2	471	8.45	8.1	22
AGR	AMB-3_2	Verla 2, Giovo	11	2	470	9.15	10.61	22.7
AGR	AMB-3_3	Cembra 1 & 3, Lisignago	10	2	486	8.06	5.78	25.7
AGR	AMB-3_4	Lisignagno 1	11	2	499	6.83	0.05	22.8
PAS	AMB-4_2	Monte Baldo near Postemon, Brentonico	11	2	1466	7.32	2.42	23.5
PAS	AMB-4_3	Postemon, Monte Baldo, Brentonico	12	2	1379	7.35	3.28	19.4
PAS	AMB-4_4	Malga Campei, Monte Baldo, Brentonico	11	2	1306	7.21	1.01	19.2
PAS	AMB-4_5	Malga Palazzo, Besenello	11	2	1533	6.96	13.72	29.1

^a^FLU: two natural pools bordering the Avisio River in the Adige Valley (i.e., seasonal accumulations of water which form in the narrow river floodplains during periods of high flow, typically in spring and summer, “pozze laterali fluviali” in [28]), VBE: four ephemeral ponds (puddles forming in wheel ruts, “raccolte d’acqua temporanee” in [28]) and artificial drainage ditches (“fossi agricoli”, [28]) in apple orchards in the Piana Rotaliana, AGR: irrigation tanks in the vineyards in Val di Cembra (concrete structures for water collection, with a perimeter of 2–6 m, “vasche agricole” in [28]), and PAS: four high elevation farm ponds on Monte Baldo, Monte Bondone and the Altopiano della Vigolana (basins for watering grazing livestock, “pozze d’alpeggio” in [28])

^b^Identification codes for each sampling site

^c^Number of *B. variegata* skin samples collected (one per individual) for microbiota analysis

^d^Number of water samples collected for water microbiota analysis. Water pH, dissolved oxygen, and water temperature were measured near the shoreline, where these animals were found

Six to 15 apparently healthy adult individuals per site were sampled between April and July 2022 (Table 1). Field workers wore sterile gloves, changing them between each toad, which was captured by hand or using a dip-net, rinsed with sterile water to remove transient bacteria, and swabbed with a GenoTube Livestock Swab (Thermo Fisher Scientific, USA), gently stroking their ventral and axillary areas for 20 s. Two water samples (200–500 ml) were collected per site from the shore and filtered with Sterivex GP Filter units (pore size 0.22 µm, Millipore cat. no. SVGPL10RC, Merck KGaA, Darmstadt, Germany) following [34]. Water temperature and dissolved oxygen were measured from the shoreline with an oximeter model Seven2Go DO (Mettler Toledo), and pH with a pH meter model Seven2Go pH/Ion meter S8 (Mettler Toledo). Site elevation was measured using Google Earth Pro version 7.3.6.9345, 2022. All samples (146 swabs and 28 filters) were transported at ambient temperature [35] to the Animal, Environmental and Antique DNA Platform at the Fondazione E. Mach (FEM) within 1 h of sampling, and then stored at − 20 °C until DNA extraction.

### Laboratory Workflow

All laboratory procedures were carried out under BSL2 biological hoods at FEM. DNA extraction from skin swabs was performed using the DNeasy Blood & Tissue Kit (Qiagen, Germany) following the Quick-Start standard protocol, including elution of extracted DNA in 200 µl Buffer AE. The lysis step was carried out overnight to maximize extraction of bacteria and fungi. Filter samples were extracted with the DNeasy PowerWater Sterivex Kit (Qiagen, Germany), following the manufacturer’s instructions (with a final elution in 100 μl of Solution EB). Each extraction batch included one negative control (sterile water only). Extracted DNA was stored at − 20 °C until amplification.

The bacterial V3-V4 fragment of 16S rRNA gene was amplified with primers 341F (CCTACGGGNGGCWGCAG) and 805R (GACTACNVGGGTWTCTAATCC) [36, 37] (expected size ~ 460 bp; [38) anchored with Illumina forward and reverse overhang adapters (https://support.illumina.com/documents/documentation/). The reaction mixture consisted of 12.5 µl of 2 × KAPA HiFi HS ReadyMix, forward and reverse primer to a final concentration of 0.32 µM, 3 µl of template DNA, and sterile H_2_O to a final volume of 25 µl. The PCR program followed the KAPA Kit protocol (31 cycles and annealing temperature of 55 °C). For fungal metataxonomy, the ITS1 region was amplified with primers ITS5 (GGAAGTAAAAGTCGTAACAAGG) and ITS2 (GCTGCGTTCTTCATCGATGC) [39] as described above. The expected size of the ITS1 fragment is usually around 250–400 bp (e.g., [40–42]. PCR reactions were carried out in a reaction mixture consisting of 10 µl of Promega Flexi Buffer 5X, forward and reverse primer to a final concentration of 0.2 µM, 1 µl of dNTP’s 10 mM each, 0.25 µl of Promega-GoTaq HS G2 5U/µl, 5 µl of MgCl_2_ 25 mM, 5 µl of template DNA and sterile H_2_O up to a volume of 50 µl. The PCR program followed the Promega Taq Kit parameters (40 cycles and annealing temperature of 55 °C). Each round of PCR included three extraction blanks and two to three PCR blanks (reaction mixture only). All amplification products were then purified with the CleanNGS Kit CNGS-0050 (CleanNA, The Netherlands) following the manufacturer’s instructions, and sequenced at the FEM Sequencing and Genotyping Platform with paired-end sequencing (2 × 300 base pairs, bp) on an Illumina Miseq (Illumina, San Diego, CA). The Illumina sequencing protocol therefore covered both target regions, based on our expected amplicon sizes. The sequencing depth per sample was 100,000 reads per sample for bacteria and 30,000 for fungi (following in part [41, 42]).

### Bioinformatics and Statistical Analysis

Raw reads processing and taxonomic assignment were performed with the R package *DADA2* V1.30.0 [43, 44] using default parameters, except for the following modifications. For the 16S bioinformatic workflow, at the *filterAndTrim* step the option trimLeft was set as c(17,21) and truncLen as c(280,250). The database used for taxonomic assignment was silva_nr99_v138.1 [45]. The initial raw reads were 21,407,900 and after bioinformatic processing 10,318,149. For the ITS1 bioinformatic workflow, primer trimming was performed with *cutadapt* by assessing all possible primer orientations to ensure complete primer removal. Taxonomic assignment was performed with the UNITE database version 9.0 (UNITE general FASTA release for eukaryotes, released on 18.07.2023; [46]). In this case, the initial dataset consisted of 8,481,680 raw reads, and after bioinformatic processing 3,683,426. At the end of both workflows, all data were converted to a *phyloseq* object [47] for further analysis. The prevalence-based method of the R package *decontam* (v1.20.0; [48]) was applied to detect and filter contaminating ASVs detected from negative controls. The number of retrieved ASVs at the end of all bioinformatic steps was 50,984 for bacteria and 7343 for fungi.

The following statistical analyses were performed with the R packages *microeco* v1.3.0 [49] and *vegan* v2.6.4 [50]. Fungal ASVs matching *Bd* (ncbi ACC ID: NR_119535.1) were identified using the UNITE-based taxonomic classification and confirmed by BLASTn [51]. Association between habitat subtypes and *Bd* detection was tested with Chi-squared test with the R package *stats* v4.3.2. As the presence of bacteria with antifungal activity has been shown to inhibit *Bd* infection, to detect bacteria with putative antifungal activity in our samples, the bacterial ASVs found in our samples were mapped onto the sequence database by Woodhams et al. [14]. Only sequences spanning the entire V3-V4 16S rRNA gene region annotated as *Bd*-inhibitory bacteria were included in the analysis (649 sequences). Skin and water ASVs were matched to the corresponding *Bd*-inhibitory sequences trimmed to the V3-V4 16S rRNA gene portion using the closed reference clustering strategy implemented in MICCA [52]; sequence identity threshold: Correlation between the presence and relative abundance of each bacterial ASV (including ASVs with putative *Bd*-inhibitory activity) and the following three variables: detection of Bd, relative abundance of *Bd*-related ASVs, and the number of *Bd*-related ASVs, was tested using Spearman’s rank correlation coefficient with the R package corrplot V0.95.

The number of ASVs shared between water and skin microbiota within and between habitat subtypes was estimated using the *microeco* function “trans_venn” after merging all libraries according to the variable (e.g., habitat subtype, habitat, and sample type). ASVs associated with significant differences in their abundance across habitat subtypes were identified with a differential abundance testing approach carried out using non-normalized data with the ALDEx2_t function implemented in *microeco* [53]. *p* values were corrected for false discovery rate using the Benjamini–Hochberg correction (significance cutoff of FDR corrected *p* values: 0.05). Heatmaps were generated using the web tool ClustVis [54] considering bacterial and fungal ASV counts normalized using relative proportions (total sum scaling, TSS) multiplied by median sequencing depth.

To estimate standard alpha diversity indices (Faith’s Phylogenetic Diversity, hereafter PD, Chao1, and Shannon diversity), bacterial and fungal libraries were rarefied to 21,598 and 2200 reads per sample, respectively, thereby losing one bacterial and six fungal libraries. Differences in alpha diversity estimates across habitat subtypes were tested using nonparametric Dunn’s Kruskal–Wallis Multiple Comparisons test with Holm *p*-value adjustment and using a significance level of 0.05.

We used linear mixed models (LMM) with the R package *lme4* [55] to investigate the impact of water temperature, dissolved oxygen, pH, and water bacterial and fungal diversity (Shannon) on alpha-diversity estimates of skin bacteria and skin fungi (as separate response variables). For these analyses, alpha diversity indices were transformed using the R package *bestNormalize*, applying Box-Cox transformation to bacterial and fungal Chao1 estimates, arcsin transformation to bacterial PD, and Ordered Quantile (ORQ) normalization (orderNorm) to bacterial Shannon. Fungal Shannon estimates did not significantly deviate from a normal distribution (Shapiro–Wilk test, *W* = 0.98658, *p* value = 0.2199) and were not transformed. All explanatory variables were scaled using the function scale in R package *dplyr* [56].

To estimate beta-diversity indices (Bray–Curtis and Jaccard), bacterial and fungal samples were first normalized using a scaling with ranked subsampling (SRS, [57]). Unconstrained ordination based on Bray–Curtis and Jaccard dissimilarity estimates was performed using non-metric multidimensional scaling (NMDS). Beta-diversity estimates were then compared across habitat subtypes using ANOSIM and permutational MANOVA (PERMANOVA) statistical tests with 999 permutations. Multivariate homogeneity of group dispersions, a necessary condition to apply the PERMANOVA test, was tested using the *vegan* betadisper function [58] in the R package *microeco*. The correlation between both bacterial and fungal beta-diversity indices with pH, temperature, and dissolved oxygen as well as water bacterial and fungal alpha diversity (Shannon) were tested with a Mantel test in R using the Spearman correlation coefficient. The same statistical test was also used to check for correlation between bacterial and fungal beta diversity estimates.

## Results

**Fig. 2:**
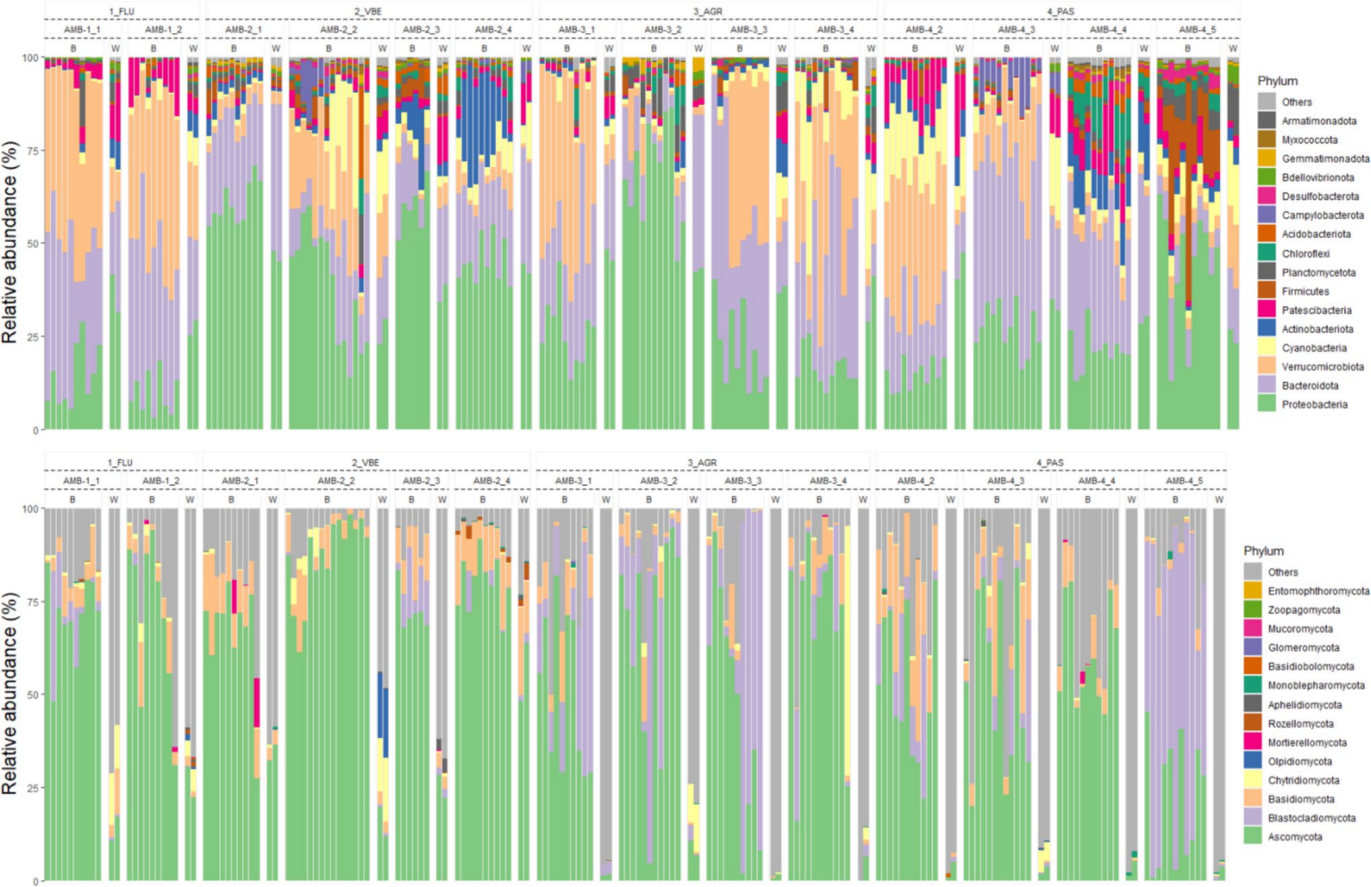
Relative abundance of Phyla of *Bombina variegata* skin (B) and water (W) microbiota. Upper panel: bacteria; lower panel: fungi. Upper bar refers to habitat subtypes (1_FLU, fluvial sites, 2_VBE, valley bottom ecotones, 3_AGR, agricultural water tanks, 4_PAS, pasture ponds); codes in the second bar to sampled sites (AMB-1_1 etc.); third bar indicates the origin of the sample: *B. variegata* skin (B) or water (W). This plot was created with the R package *ggplot2* and formatted using *GIMP* v2.10.18 (The GIMP Development Team (2019). GIMP. Retrieved from https://www.gimp.org)

Overall, this study of *B. variegata* skin microbiota detected 88 microbial Phyla, including 74 bacteria and 14 fungi. The most common bacterial Phyla in both skin and water communities were Proteobacteria, Bacteroidota and Verrucomicrobiota, followed by Cyanobacteria, Actinobacteriota, and Patescibacteria (see Fig. 2). The most common fungal Phylum in skin swabs was Ascomycota, followed by Basidiomycota (Fig. 2). Of note, while taxonomic classification at Phylum level in skin samples exceeded 75% of sequence reads in most cases (Fig. 2), the majority (median 82.5%; range: 14.4–99.4%) of fungal reads in water samples could not be classified even at this high taxonomic level (Fig. 2).

Despite sampled toads lacking noticeable symptoms of pathogen infection, the taxonomic classification of fungi in skin samples led to the identification of 26 ASVs matching *Bd* (Table S1), which were not detected in our water samples. The 18 *Bd*-positive skin samples (12% of collected swabs) spanned all four habitat subtypes (8/14 sites): five were from FLU (AMB_1_1, AMB_1_2), three from PAS (AMB_4_3, AMB_4_5), three VBE (AMB_2_2), and seven from AGR (AMB_3_2, AMB_3_3, AMB_3_4). Overall, *Bd* detection was not statistically associated with any of the studied habitat subtypes (Pearson’s chi-squared test, *p* value = 0.08987).

Using publicly available databases [14], we identified 63 bacterial taxa (ASVs) with putative anti-*Bd* activity (Table S2). Almost a third of these were classified as *Pseudomonas* spp. (18 ASVs). Other genera represented by three or more distinct ASVs were *Acinetobacter* (5 ASVs), *Stenotrophomonas* (5 ASVs), *Chryseobacterium* (3 ASVs), *Flavobacterium* (3 ASVs), and members of the family Aeromonadaceae (3 ASVs). These taxa were found in both water and skin samples, from all four habitats. However, the relative abundance or presence of putative *Bd*-inhibitory ASVs did not show any correlation with the detection of *Bd* in skin samples, the number of *Bd* related ASVs, or the cumulative relative abundance of *Bd* related ASV (Spearman correlation coefficient ranging from − 0.15 to + 0.15 considering both relative abundance and presence of putative *Bd*-inhibitory ASVs; Figure S1, Table S2). Moreover, extending this analysis to the entire set of bacterial ASVs identified in this study did not highlight significant correlations between abundance of skin bacterial ASVs and detection of *Bd* in skin samples, the number of *Bd* related ASVs or the cumulative relative abundance of *Bd* related ASVs (Spearman correlation coefficient ranging from −0.21 to + 0.43).

The comparison of bacterial and fungal ASVs found in skin and water in each habitat subtype revealed that the microbiota of water and skin communities were quite distinct (Figure S2A, B), with a relatively low number of shared bacterial and fungal ASVs. Nonetheless, these accounted for more than half of generated reads in each habitat (range: 55.5 to 80.9% for bacteria; 60.2 to 76.8% for fungi). Skin microbial communities, defined as ASVs detected only from skin samples in each habitat, included 32,022 bacterial and 2774 fungal ASVs (Figure S2C, D) representing 69.57% and 68.60% of identified bacterial and fungal ASVs. Of these, only about 1% (e.g., 302 bacterial ASVs and 39 fungal ASVs) were detected in at least one sample from each habitat subtype (Figure S2C, D). However, again, these few ASVs shared between skin and water accounted for approximately 63% and 31% of total reads sequenced from bacterial and fungal skin communities, respectively. The comparison of water and skin microbiota using bacterial ASVs collapsed at Phylum level revealed that while a significant fraction of identified Phyla was only detected in skin or water samples (with the number of non-shared Phyla ranging from 12/66 for AGR to 15/50 for FLU), these private Phyla accounted for less than 1% of sequence reads, highlighting that shared Phyla were by far the most abundant.

**Fig. 3:**
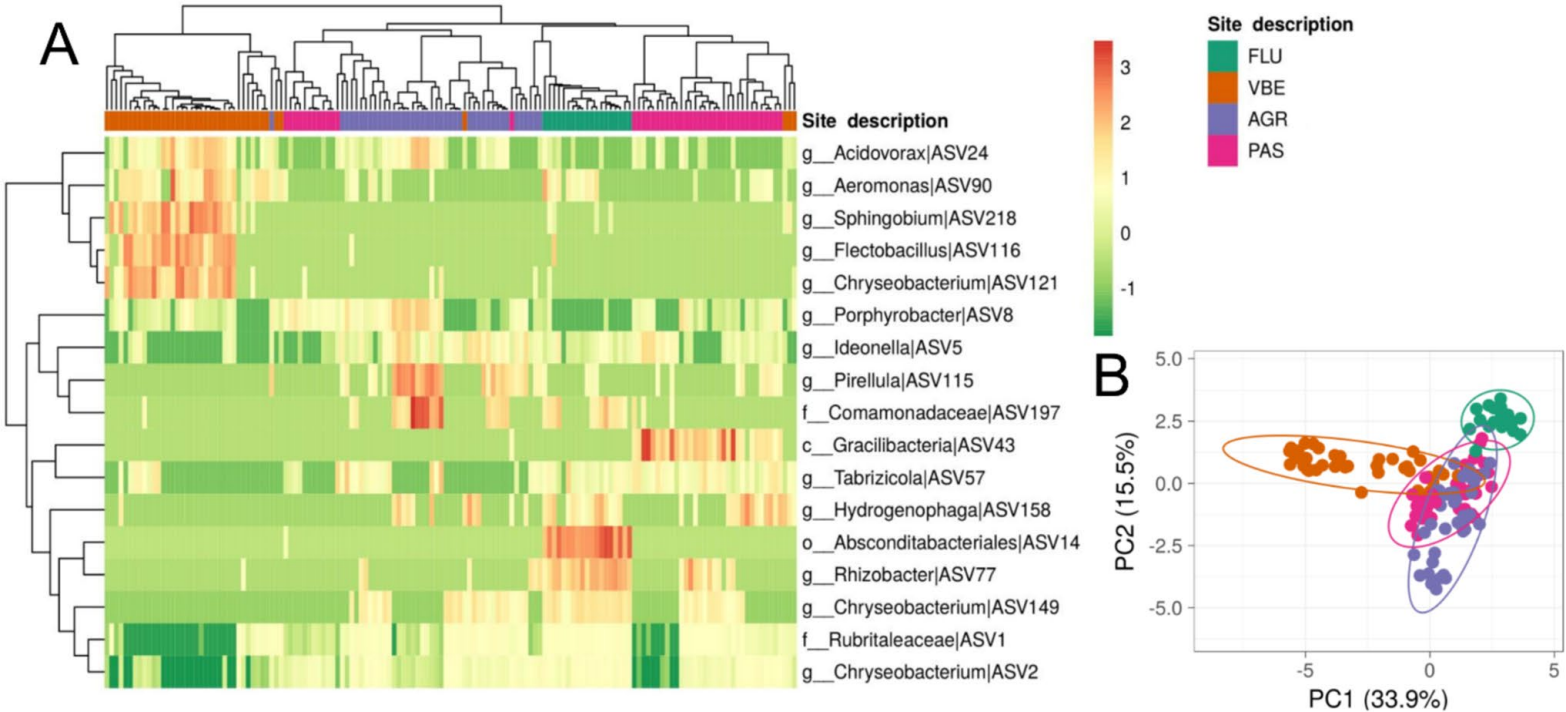
Heatmap (**A**) and PCA (**B**) of the 17 bacterial taxa of *B. variegata* skin microbiota with significant differences in abundance across four habitat subtypes (FLU, VBE, AGR, and PAS). To generate both A and B, original values of ASV abundance were ln(*x* + 1)-transformed. In (**A**), rows were centered, unit variance scaling was applied to rows, and both rows and columns were clustered using correlation distance and average linkage (74 rows, 147 columns). Abundance is represented with colors from green (rank − 1; least abundant) to red (rank 3; most abundant). For the PCA, unit variance scaling was applied to rows, and singular value decomposition (SVD) with imputation was used to calculate principal components. *X*- and *Y*-axes show principal components 1 and 2 that explain 33.9% and 15.5% of the total variance, respectively. Prediction ellipses are such that with probability 0.95, a new observation from the same group will fall inside the ellipse. This plot was created with ClustVis and formatted using *GIMP* v2.10.18 (The GIMP Development Team (2019). GIMP. Retrieved from https://www.gimp.org)

Overall, 17 bacterial ASVs showed significant differences in abundance in skin microbial communities among habitat subtypes (Fig. 3, Table S3). These differentially abundant taxa suggested a clear separation between VBE and the other habitats (Fig. 3A) as well as the separation of FLU from AGR and PAS (Fig. 3B). Of note, ten, five, and three taxa found in FLU skin samples showed significant differences in abundance compared to the same taxa in VBE, PAS and AGR samples, respectively (Table S3, Fig. 3A). Conversely, only three fungal ASVs classified as *Aureobasidium* (Ascomycota), *Claviceps* (Ascomycota), and *Catenaria* (Blastocladiomycota) showed significant differences across habitat subtypes (Table S3).

**Fig. 4:**
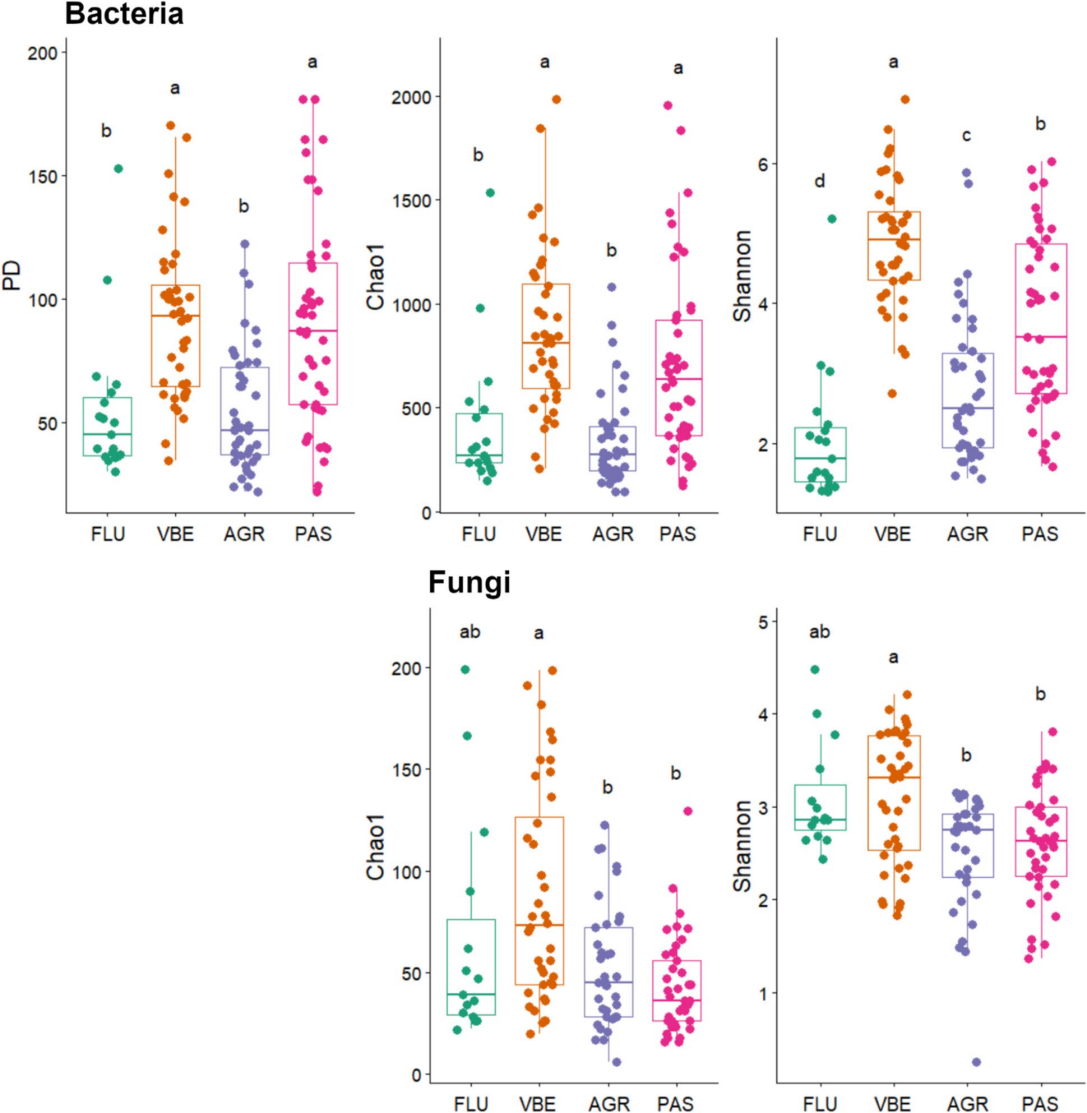
Alpha diversity estimates (PD: Faith’s phylogenetic diversity, Chao1 and Shannon) of *B. variegata* skin microbiota across four habitat subtypes (FLU, VBE, AGR, and PAS) in the province of Trento, Italy. Upper panel: bacteria; lower panel: fungi. This plot was created with the R package *ggplot2* and formatted using *GIMP* v2.10.18 (The GIMP Development Team (2019). GIMP. Retrieved from https://www.gimp.org)

The alpha diversity estimates calculated from *B. variegata* skin swabs varied between habitat subtypes (Fig. 4), with the skin microbiota of individuals from FLU and AGR showing significantly lower bacterial PD and Chao1 richness estimates than skin samples collected from the other two habitats (VBE, PAS). Shannon diversity estimates for bacterial communities were significantly different across all four habitats, with VBE having the highest diversity followed by PAS, AGR, and FLU (Dunn’s Kruskal–Wallis Multiple Comparisons, *p* < 0.05; Fig. 4, Table S4). In addition, for each of the three diversity indices, significant differences were detected between skin bacterial communities hosted by animals from different sampling sites within habitat subtypes AGR and PAS, but not FLU and VBE (Dunn’s Kruskal–Wallis Multiple Comparisons, *p* < 0.05; Figure S3).

Fungal richness and diversity, considerably lower than those observed for bacteria, also varied significantly between habitat subtypes, with skin mycobiota of VBE toads characterized by higher Chao1 and Shannon diversity estimates than those sampled in PAS and AGR (Dunn’s Kruskal–Wallis Multiple Comparisons, *p* < 0.05; Fig. 4, Table S4). Moreover, similarly to what was observed for bacterial communities, Chao1 and Shannon diversity estimates of skin fungal communities varied between sampling sites within each habitat subtype except PAS, for which we found no significant difference for Shannon diversity estimates (Dunn’s Kruskal–Wallis Multiple Comparisons, *p* < 0.05; Figure S3).

Linear mixed models indicated that observed variation in skin bacterial richness (Chao1) and PD across habitats and sampling sites was negatively associated with pH and positively associated with measured levels of dissolved oxygen (Table S5). Notably, the same associations were not detected when bacterial alpha diversity was estimated as Shannon diversity, suggesting that rare or low abundance taxa might be vulnerable to these abiotic changes. Interestingly, variation in the same abiotic factors was not associated with changes in fungal diversity (both indices, Table S5). Instead, the observed Shannon diversity, but not richness (Chao1), of fungal communities was negatively correlated with variation in temperature across the investigated habitats and sampling sites.

**Fig. 5:**
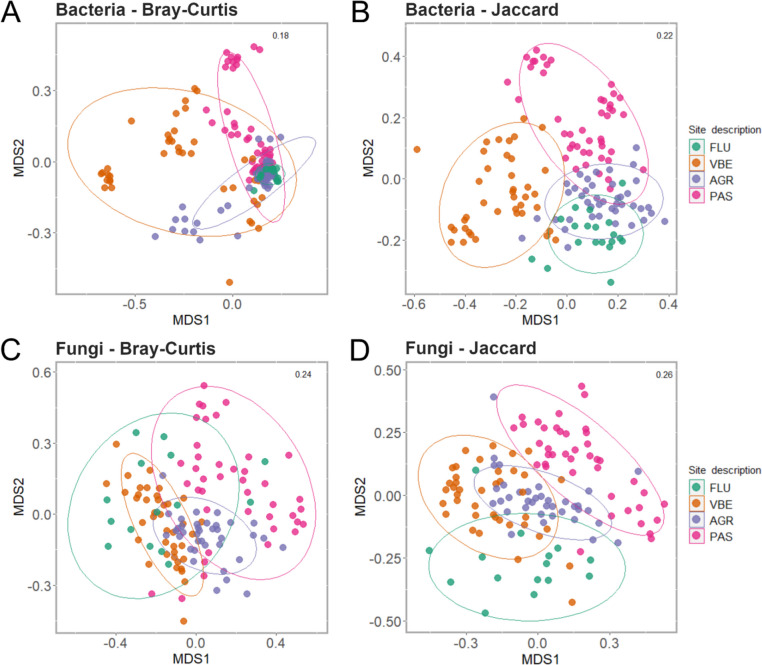
Beta diversity estimates (left: Bray–Curtis; right: Jaccard) of *B. variegata* skin microbiota across the four habitat subtypes (FLU, VBE, AGR, and PAS). Upper panel: bacteria; lower panel: fungi. This plot was created with the R package *ggplot2* and formatted using *GIMP* v2.10.18 (The GIMP Development Team (2019). GIMP. Retrieved from https://www.gimp.org)

The composition of skin and water microbiota, as measured by Bray–Curtis and Jaccard dissimilarity indices, varied significantly between habitat subtypes (Fig. 5: skin samples; Figure S4: skin and water samples). For both indices, the four subtypes were characterized by differences in dispersion of bacterial skin microbiota (BETADIPER, Bray–Curtis *F*: 40.50, *p* value ≤ 0.001; Jaccard *F*: 6.2695, *p* value ≤ 0.001), as highlighted by the close clustering of FLU bacterial communities compared to the widespread distribution of VBE and PAS communities (Fig. 5A, B). However, pairwise dissimilarity estimates and clustering of samples according to both indices showed that skin bacterial communities of animals collected from the same habitat were more similar to one another than to those of other habitats (ANOSIM, Bray–Curtis *R*: 0.248, *p* value ≤ 0.001; Jaccard *R*: 0.601, *p* value ≤ 0.001). Consistent with this result, both dissimilarity estimates highlighted significant differences across skin bacterial communities hosted by animals collected from different habitat subtypes (PERMANOVA, Bray–Curtis *R*^2^: 0.183, *F*: 20.03, *p* value ≤ 0.001; Jaccard *R*^2^: 0.075, *F*: 4.50, *p* value ≤ 0.001; Tables S6, S7 and Fig. 5). In particular, our results show that VBE is clearly differentiated from the other subtypes, especially in terms of relative abundances of skin bacterial taxa, followed closely by FLU (Table S7). In terms of presence/absence data of bacterial taxa, however, all subtypes appear to be more equally distinct (Table S7). Nevertheless, a significant fraction of variation in dissimilarity estimates was found to be associated with differences across sampling sites within each habitat subtype (PERMANOVA, Bray–Curtis *R*^2^: 0.414, *F*: 13.56, *p* value ≤ 0.001; Jaccard *R*^2^: 0.190, *F*: 3.40, *p* value ≤ 0.001; Fig. 5, Table S6), with skin bacterial communities found in VBE and PAS being characterized by the highest differentiation across sampling sites according to both indices (Fig. 5).

The ordination of skin fungal communities based on Bray–Curtis and Jaccard dissimilarity indices grouped the skin samples into distinct clusters corresponding to the four habitat subtypes. Again, separation between subtypes was evident when ecological dissimilarity between communities was estimated with the Bray–Curtis (ANOSIM *R*: 0.3643, *p* value ≤ 0.001; PERMANOVA *R*^2^: 0.126, *F*: 3.450, *p* value ≤ 0.001; Table S6) and Jaccard indices (ANOSIM *R*: 0.5204, *p* value ≤ 0.001; PERMANOVA *R*^2^: 0.074, *F*: 3.450, *p* value ≤ 0.001 Fig. 5C, D and Figure S4C, D; Tables S6, S7). Furthermore, as reported above for bacterial communities, a large proportion of variation in diversity estimates was associated with differences across sampling sites considered in each habitat subtype (PERMANOVA, Bray–Curtis *R*^2^: 0.231, *F*: 4.27, *p* value ≤ 0.001; Jaccard *R*^2^: 0.154, *F*: 2.37, *p* value ≤ 0.001; Table S6, Fig. 5C, D). Notably, both beta diversity indices show an association between skin communities and water communities from the same site for both bacteria and fungi (PERMANOVA *p* value ≤ 0.001 for both sample type and interaction term between site code: sample type; Figure S4; Table S6), although overlap in community composition is higher when dissimilarity was estimated with Jaccard index (Figure S4; Table S6).

Mantel tests highlighted that water temperature had the strongest correlation with observed differences in bacterial (Spearman correlation: Bray–Curtis *R* = 0.53, *p* = 0.002 and Jaccard *R* = 0.43, *p* = 0.001) and fungal diversity estimates (Spearman correlation: Bray–Curtis *R* = 0.31, *p* = 0.002 and Jaccard *R* = 0.30, *p* = 0.002) (see Table S8). Additionally, we found evidence of correlation between dissolved oxygen and skin bacterial diversity (Spearman correlation: Bray–Curtis *R* = 0.25, *p* = 0.002 and Jaccard *R* = 0.20, *p* = 0.001) as well as skin fungal diversity (Spearman correlation: Bray–Curtis *R* = 0.20, *p* = 0.002 and Jaccard *R* = 0.27, *p* = 0.002), while other parameters showed much lower correlation coefficients (Table S8). It is interesting to note, however, that the composition of bacterial and fungal diversity appears to be poorly correlated, but that the correlation is stronger when the comparison between pairs of samples is made using presence/absence data. (Mantel test, Bray–Curtis *R*: 0.08093, *p* value: 0.037; Jaccard *R*: 0.1583; *p* value: 1*e* − 04).

## Discussion

Skin microbial communities may be fundamental for amphibians to adapt to challenging environments such as polluted or highly seasonal water bodies. This study aimed, for the first time, to characterize the skin microbiota of the yellow-bellied toad*,* a locally threatened amphibian, across a variety of habitat subtypes. Despite the potential conservation relevance and the number of papers on bacterial skin communities, this is one of the first amphibian microbiota studies focusing on both bacterial *and* fungal skin diversity. Overall, although core taxa for both skin bacterial and fungal communities were similar to those previously reported separately, we found an unexpected association between the diversity indices of both microbiota components. These indices varied significantly across habitat subtypes, and there were strong associations between skin bacteria and fungi and those of the water from each habitat subtype, as well as with water parameters.

The main bacterial Phyla characterizing the skin microbiota of the yellow-bellied toad, Proteobacteria, Bacteroidota, and Verrucomicrobiota also dominate the skin microbial community of other amphibian species (e.g., *B. orientalis* [59], *Thoropa taophora* [60], and five species of the *Pseudoeurycea* genus [61]). Since several Proteobacteria species are known to produce anti-*Bd* metabolites, the abundance of this taxon on *B. variegata* skin may explain the apparently low susceptibility of this toad to *Bd*, as has been suggested for another species from warmer climate (*T. taophora* from Brazil [60]). The high prevalence of Ascomycota and Basidiomycota is also in agreement with previous studies on amphibian skin mycobiota [9, 60, 61]. Interestingly, most fungal taxa found in our water samples were classified as “unknown” (i.e., not present in the standard database for metataxonomy of fungi, UNITE). The lack of data on wetland fungal diversity has in fact been noted by several authors [62]. Considering the clear association of wetland fungal communities with that of the yellow-bellied toad, and by extension, to all amphibian species with life stages dependent on water bodies, we suggest further research focused specifically on water mycobiota, in order to deepen our understanding of its impact on freshwater organisms.

Worryingly, for the first time in the Province of Trento, we detected ASVs from amphibian skin swabs showing 100% identity with the nucleotide sequences of *Bd* reference specimens (NCBI ACC: NR_119535.1). Several individuals in each of the four habitat subtypes were positive for *Bd*, but there was no clear pattern of association of *Bd* presence with subtype. All individuals with these *Bd* ASVs appeared asymptomatic. The apparent lack of symptoms of the sampled individuals to *Bd* infection could be due to the presence of 63 putative *Bd*-inhibitory bacterial taxa. This number is also likely to be an underestimate of the richness of inhibitory taxa since most of the reference sequences in Woodhams et al. [14] were obtained from other continents (e.g., North America, New Zealand) and only 89 were from Europe (Switzerland). On the other hand, no clear correlation was found between presence or relative abundance of *Bd*-related ASVs and bacterial ASVs, including those matching putative *Bd*-inhibitory bacteria. Nevertheless, we should note that the low number of *Bd*-infected samples, their distribution among different habitat subtypes, and the high bacterial diversity among habitat subtypes made the identification of clear correlations between the incidence of this pathogenic fungus and changes in specific bacterial taxa particularly challenging. Moreover, *Bd* virulence has been shown to depend on strain (e.g., [63]), and recent reports suggest that European strains may be less virulent than those of other continents, and possibly even protect amphibians from colonization with more lethal ones [64]. This could explain why wild populations of yellow-bellied toad appear to be highly tolerant to this pathogen. However, as landscape context has been identified as a major aspect shaping *Bd* distribution, changes in land use and water quality could alter this precarious coexistence in the near future [65]. Therefore, our result, if confirmed, is highly relevant for amphibian conservation in northern Italy [32].

In agreement with previous studies (e.g., two salamander genera, *Ensatina* and *Batrachoseps* [6]), our skin samples showed a high proportion of unique ASVs (not found in water samples; Figure S2), despite the toads’ skin being in direct contact with the water microbiota, suggesting that skin microbiota composition is highly selected by the host. In fact, only a low number of ASVs were shared between skin and water, although shared taxa were present with a high relative abundance in both sample types (Figure S2). Similar numbers have been reported for other amphibian species (e.g., *Eleutherodactylus johnstonei* [21]). Nonetheless, the overall *B. variegata* skin microbial community appears to be largely composed of the relatively few, but highly abundant, ASVs that are also found in their environment, thus supporting the influence of habitat subtype on skin microbiota and highlighting once more the importance of habitat conservation for these sensitive species. As both bacterial and fungal communities have shown a strong association with water microbiota communities, we suggest that conservation strategies for *B. variegata*, and amphibians more in general, include environmental microbiota and the impact of anthropogenic activities in these environments.

Our skin samples highlighted several differentially abundant bacterial taxa between habitats, including Gammaproteobacteria as well as Bacteroidia, Alphaproteobacteria, Gracilibacteria, Planctomycetes, and Verrucomicrobiae. These taxa present different characteristics in terms of habitat preferences and metabolism; therefore, further investigation will be needed to understand the significance of this composition. In addition, both alpha and beta-diversity estimates for skin bacteria and fungi were significantly different for individuals living in distinct habitat subtypes, confirming recently published results that found, for other amphibian species, variations in skin community composition according to habitat type (e.g., *T. thaofora* [60], *Anaxyrus boreas* [17], and eight other species [51]). In particular, individuals of VBE sites showed a higher diversity for both microbial communities and both indices. Several authors have shown the association between a rich and diverse microbiota and healthier individuals [e.g., 66]. Therefore, we argue that *B. variegata* populations living in ephemeral ponds along the bottom of Adige Valley are potentially healthier and more resistant to *Bd* [67], although a more comprehensive study with a higher number of samples will be needed to test this hypothesis, as well as to provide relevant insights on *Bd* presence in the area (i.e., which strain is present).

Alpha and beta diversity indices for bacterial and fungal communities also showed strong correlations with the environmental parameters considered here. For example, bacterial richness (alpha diversity) was positively correlated with dissolved oxygen in the water and negatively correlated with water pH. While the negative impact of water pH confirms results from other studies [25, 68], the positive effect of dissolved oxygen was less expected (i.e., [68] found a moderate negative correlation for this variable) but could help explain the lower diversity found in individuals sampled in ponds subjected to eutrophication (i.e., pasture ponds, PAS). Both bacterial and fungal beta diversity were positively correlated with water temperature and, although less strongly, with dissolved oxygen. Again, the positive effect of temperature we found is in agreement with previous studies, although available only for bacterial communities [25, 68], while the positive influence of dissolved oxygen represents a novel finding. Therefore, for future *B. variegata* populations conservation planning, our results support the need to preserve and possibly increase freshwater sites where waters are moderately warm and oxygenated, and eutrophication is limited.

Since this is a relatively unexplored field, our results are timely as they show more specific associations between water parameters and skin microbiota composition and richness. García-Sánchez and colleagues [61] reported that fungal skin community composition of amphibians appears to be even more strongly influenced by climatic conditions than that of bacterial communities, which they found more dependent on host phylogenetic relatedness. Overall, the association of skin and environment microbial communities seems so strong that they could be considered meta-communities, as recently suggested by Leonhardt et al. [21]. For this reason, we support a holistic approach to the study of amphibian microbiota, that includes both bacterial and fungal communities of both host and environment.

## Supplementary Information

Below is the link to the electronic supplementary material.

Supplementary file1 (DOCX 2654 KB)

Supplementary file2 (XLSX 87 KB)

## Data Availability

The datasets generated and analyzed during the current study have been submitted to the EMBL Database (Project Accession: PRJEB81466), and will be made publicly available upon acceptance of the article.

## References

[1] Cordier JM, Aguilar R, Lescano JN, Leynaud GC, Bonino A, Miloch D, Loyola R, Nori J (2021) A global assessment of amphibian and reptile responses to land-use changes. Biol Conserv 253:108863. 10.1016/j.biocon.2020.108863

[2] Jiménez RR, Sommer S (2017) The amphibian microbiome: natural range of variation, pathogenic dysbiosis, and role in conservation. Biodivers Conserv 26:763–786. 10.1007/s10531-016-1272-x

[3] Bernardo-Cravo AP, Schmeller DS, Chatzinotas A, Vredenburg VT, Loyau A (2020) Environmental factors and host microbiomes shape host–pathogen dynamics. Trends Parasitol 36(7):616–633. 10.1016/j.pt.2020.04.010 32402837 10.1016/j.pt.2020.04.010

[4] Woodhams DC, McCartney J, Walke JB, Whetstone R (2023) The adaptive microbiome hypothesis and immune interactions in amphibian mucus. Dev Comp Immunol 145:104690. 10.1016/j.dci.2023.104690 37001710 10.1016/j.dci.2023.104690 PMC10249470

[5] Hernández-Gómez O, Kimble SJA, Briggler JT, Williams RN (2017) Characterization of the cutaneous bacterial communities of two giant salamander subspecies. Microb Ecol 73:445–454. 10.1007/s00248-016-0859-9 27677893 10.1007/s00248-016-0859-9

[6] Bird AK, Prado-Irwin SR, Vredenburg VT, Zink AG (2018) Skin microbiomes of california terrestrial salamanders are influenced by habitat more than host phylogeny. Front Microbiol 9:442. 10.3389/fmicb.2018.00442 29593686 10.3389/fmicb.2018.00442 PMC5861191

[7] Hartmann AM, McGrath-Blaser SE, Colón-Piñeiro Z, Longo AV (2023) Ontogeny drives shifts in skin bacterial communities in facultatively paedomorphic salamanders. Microbiology 169(10):001399. 10.1099/mic.0.001399 37815535 10.1099/mic.0.001399 PMC10634365

[8] Bletz MC, Perl RB, Bobowski BT et al (2017) Amphibian skin microbiota exhibits temporal variation in community structure but stability of predicted Bd-inhibitory function. ISME J 11(7):1521–1534. 10.1038/ismej.2017.41 28387770 10.1038/ismej.2017.41 PMC5520157

[9] Estrada A, Hughey MC, Medina D et al (2019) Skin bacterial communities of neotropical treefrogs vary with local environmental conditions at the time of sampling. PeerJ 7:e7044. 10.7717/peerj.7044 31275740 10.7717/peerj.7044 PMC6590418

[10] Kueneman JG, Bletz MC, Becker M et al (2022) Effects of captivity and rewilding on amphibian skin microbiomes. Biol Conserv 271:109576. 10.1016/j.biocon.2022.109576

[11] Woodhams DC, Barnhart KL, Bletz MC, et al (2018) *Batrachochytrium*: biology and management of amphibian chytridiomycosis. eLS, 1–18. 10.1002/9780470015902.a0027207

[12] Woodhams DC, LaBumbard BC, Barnhart KL et al (2018) Prodigiosin, violacein, and volatile organic compounds produced by widespread cutaneous bacteria of amphibians can inhibit two Batrachochytrium fungal pathogens. Microb Ecol 75:1049–1062. 10.1007/s00248-017-1095-7 29119317 10.1007/s00248-017-1095-7

[13] Walke JB, Becker MH, Loftus SC et al (2015) Community structure and function of amphibian skin microbes: an experiment with bullfrogs exposed to a chytrid fungus. PLOSOne 10(10):e0139848. 10.1371/journal.pone.0139848 10.1371/journal.pone.0139848 PMC4596541 26445500

[14] Woodhams DC, Alford RA, Antwis RE et al (2015) Antifungal isolates database of amphibian skin-associated bacteria and function against emerging fungal pathogens. Ecology 96(2):595–595. 10.1890/14-1837.1

[15] Fieschi-Méric L, Van Leeuwen P, Denoël M, Lesbarrères D (2023) Encouraging news for in situ conservation: translocation of salamander larvae has limited impacts on their skin microbiota. Mol Ecol 32(12):3276–3289. 10.1111/mec.16914 36872055 10.1111/mec.16914

[16] Kearns PJ, Fischer S, Fernández-Beaskoetxea S et al (2017) Fight fungi with fungi: antifungal properties of the amphibian mycobiome. Front Microbiol 8:2494. 10.3389/fmicb.2017.02494 29312201 10.3389/fmicb.2017.02494 PMC5735112

[17] Alexiev A, Melie T, Martindale R, Delacey C, Quandt CA, McKenzie VJ (2023) Mr. Toad’s wild fungi: fungal isolate diversity on Colorado boreal toads and their capacity for pathogen inhibition. Fungal Ecol 66:101297. 10.1016/j.funeco.2023.101297 38487623 10.1016/j.funeco.2023.101297 PMC10938945

[18] Stupar M, Savković Ž, Breka K, Stamenković S, Krizmanić I, Vukojević J, Grbić ML (2023) A variety of fungal species on the green frogs’ skin (*Pelophylax esculentus* complex) in South Banat. Microbial Ecol 86(2):859–871. 10.1007/s00248-022-02135-0 10.1007/s00248-022-02135-0 36322177

[19] McKnight DT, Huerlimann R, Bower DS, Schwarzkopf L, Alford RA, Zenger KR (2022) The interplay of fungal and bacterial microbiomes on rainforest frogs following a disease outbreak. Ecosphere 13(7):e4037. 10.1002/ecs2.4037

[20] Sun D, Herath J, Zhou S, Ellepola G, Meegaskumbura M (2023) Associations of Batrachochytrium dendrobatidis with skin bacteria and fungi on Asian amphibian hosts. ISME Communications 3(1):123. 10.1038/s43705-023-00332-7 37993728 10.1038/s43705-023-00332-7 PMC10665332

[21] Leonhardt F, Keller A, Arranz Aveces C, Ernst R (2023) From alien species to alien communities: host- and habitat-associated microbiomes in an alien amphibian. Microb Ecol 86:2373–2385. 10.1007/s00248-023-02227-5 37233803 10.1007/s00248-023-02227-5 PMC10640505

[22] Ramírez-Barahona S, González-Serrano FM, Martínez-Ugalde E, Soto-Pozos A, Parra-Olea G, Rebollar EA (2023) Host phylogeny and environment shape the diversity of salamander skin bacterial communities. Anim microbiom 5:52. 10.1186/s42523-023-00271-7 10.1186/s42523-023-00271-7 PMC10571319 37828573

[23] Hernández-Gómez O, Hua J (2023) From the organismal to biosphere levels: environmental impacts on the amphibian microbiota. FEMS Microbiology Reviews 47(1):fuad002. 10.1093/femsre/fuad002 36725211 10.1093/femsre/fuad002

[24] Kueneman JG, Bletz MC, McKenzie VJ, Becker CG, Joseph MB, Abarca JG (2019) Community richness of amphibian skin bacteria correlates with bioclimate at the global scale. Nat Ecol Evol 3(3):381–389. 10.1038/s41559-019-0798-1 30778181 10.1038/s41559-019-0798-1

[25] Chen H, Huang Y, Pang G, Cui Z, Wu Z, Huang H (2023) Ecological factors and anthropogenic disturbance may restructure the skin microbiota of Maoershan hynobiids (*Hynobius maoershanensis*). Diversity 15(8):932

[26] Sindaco R, Doria G, Razzetti E, Bernini F (eds) (2006) Atlante degli anfibi e rettili d'Italia/ Atlas of Italian amphibians and reptiles. Societas Herpetologica Italica, Edizioni Polistampa, Firenze

[27] IUCN SSC Amphibian Specialist Group (2022) *Bombina variegata*. The IUCN Red List of Threatened Species. e.T77970289A77954662 10.2305/IUCN.UK.2022-1.RLTS.T77970289A77954662.en Accessed 2023 November 30.

[28] Endrizzi S, Trenti M, Anderle M, Roner L, Sartori M, Romano A, Pedrini P (2023) Il monitoraggio dell’ululone dal ventre giallo (*Bombina variegata* Linnaeus, 1758) in Trentino. Studi Trentini di Scienze Naturali 102:23–36

[29] D’Amen M, Bombi P (2009) Global warming and biodiversity: evidence of climate-linked amphibian declines in Italy. Biol Conserv 142(12):3060–3067. 10.1016/j.biocon.2009.08.004

[30] Sztatecsny M, Glaser F (2011) From the eastern lowlands to the western mountains: first records of the chytrid fungus *Batrachochytrium dendrobatidis* in wild amphibian populations from Austria. Herpetol J 21(1):87–90

[31] Oswald P, Rodríguez A, Bourke J et al (2020) Locality, time and heterozygosity affect chytrid infection in yellow-bellied toads. Dis Aquat Organ 142:225–237. 10.3354/dao03543 33331290 10.3354/dao03543

[32] Costa A, Dondero L, Allaria G et al (2021) Modelling the amphibian chytrid fungus spread by connectivity analysis: towards a national monitoring network in Italy. Biodivers Conserv 30:2807–2825. 10.1007/s10531-021-02224-5

[33] Wagner N, Neubeck C, Guicking D et al (2017) No evidence for effects of infection with the amphibian chytrid fungus on populations of yellow-bellied toads. Dis Aquat Organ 123(1):55–65. 10.3354/dao03090 28177293 10.3354/dao03090

[34] Zanovello L, Girardi M, Marchesini A et al (2023) A validated protocol for eDNA-based monitoring of within-species genetic diversity in a pond-breeding amphibian. Sci Rep 13:4346. 10.1038/s41598-023-31410-4 36928612 10.1038/s41598-023-31410-4 PMC10020426

[35] Lauber CL, Zhou N, Gordon JI, Knight R, Fierer N (2010) Effect of storage conditions on the assessment of bacterial community structure in soil and human-associated samples. FEMS microbiol lett 307(1):80–86. 10.1111/j.1574-6968.2010.01965.x 20412303 10.1111/j.1574-6968.2010.01965.x PMC3148093

[36] Klindworth A, Pruesse E, Schweer T et al (2013) Evaluation of general 16S ribosomal RNA gene PCR primers for classical and next-generation sequencing-based diversity studies. Nucleic Acids Res 41(1):e1–e1. 10.1093/nar/gks808 22933715 10.1093/nar/gks808 PMC3592464

[37] Apprill A, McNally S, Parsons R, Weber L (2015) Minor revision to V4 region SSU rRNA 806R gene primer greatly increases detection of SAR11 bacterioplankton. Aquat Microb Ecol 75(2):129–137. 10.3354/ame01753

[38] Onywera H, Meiring TL (2020) Comparative analyses of ion torrent V4 and Illumina V3–V4 16S rRNA gene metabarcoding methods for characterization of cervical microbiota: taxonomic and functional profiling. Sci Afr 7:e00278. 10.1016/j.sciaf.2020.e00278

[39] White TJ, Bruns T, Lee SJWT, Taylor J (1990) Amplification and direct sequencing of fungal ribosomal RNA genes for phylogenetics. PCR Protocols: Guide Methods Appl 18(1):315–322

[40] Siddique AB, Albrectsen BR, Ilbi H, Siddique AB (2022) Optimization of protocol for construction of fungal ITS amplicon library for high-throughput illumina sequencing to study the mycobiome of aspen leaves. Appl Sci 12(3):1136. 10.3390/app12031136

[41] Ramakodi MP (2021) Effect of amplicon sequencing depth in environmental microbiome research. Curr Microbiol 78:1026–1033. 10.1007/s00284-021-02345-8 33537885 10.1007/s00284-021-02345-8

[42] Tedersoo L, Bahram M, Zinger L, Nilsson RH, Kennedy PG, Yang T, Anslan S, Mikryukov V (2022) Best practices in metabarcoding of fungi: from experimental design to results. Mol Ecol 31(10):2769–2795. 10.1111/mec.16460 35395127 10.1111/mec.16460

[43] Callahan BJ, McMurdie PJ, Rosen MJ, Han AW, Johnson AJA, Holmes SP (2016) DADA2: High-resolution sample inference from Illumina amplicon data. Nat Methods 13(7):581–583. 10.1038/nmeth.3869 27214047 10.1038/nmeth.3869 PMC4927377

[44] Callahan BJ, Sankaran K, Fukuyama JA, McMurdie PJ, Holmes SP (2016) Bioconductor workflow for microbiome data analysis: from raw reads to community analyses. F1000Res 5:1492. 10.12688/2Ff1000research.8986.2 27508062 10.12688/f1000research.8986.1 PMC4955027

[45] Quast C, Pruesse E, Yilmaz P et al (2013) The SILVA ribosomal RNA gene database project: improved data processing and web-based tools. Nucl Acids Res 41(D1):D590–D596. 10.1093/nar/gks1219 23193283 10.1093/nar/gks1219 PMC3531112

[46] Abarenkov K, Nilsson RH, Larsson KH et al (2024) The UNITE database for molecular identification and taxonomic communication of fungi and other eukaryotes: sequences, taxa and classifications reconsidered. Nucl Acids Res 52(D1):D791–D797. 10.1093/nar/gkad1039 37953409 10.1093/nar/gkad1039 PMC10767974

[47] McMurdie PJ, Holmes S (2013) phyloseq: an R package for reproducible interactive analysis and graphics of microbiome census data. PLoS ONE 8(4):e61217. 10.1371/journal.pone.0061217 23630581 10.1371/journal.pone.0061217 PMC3632530

[48] Davis NM, Proctor DM, Holmes SP, Relman DA, Callahan BJ (2016) Simple statistical identification and removal of contaminant sequences in marker-gene and metagenomics data. Microbiome 10.1101/221499 10.1186/s40168-018-0605-2 PMC6298009 30558668

[49] Liu C, Cui Y, Li X, Yao M (2021) microeco: an R package for data mining in microbial community ecology. FEMS Microbiol Ecol 97(2):fiaa255. 10.1093/femsec/fiaa255 33332530 10.1093/femsec/fiaa255

[50] Oksanen J, Simpson G, Blanchet F et al (2022) _vegan: Community Ecology Package_. R package version 2.6–4. 2022 October 11. https://cran.r-project.org/package=vegan

[51] Medina D, Hughey MC, Walke JB et al (2019) Amphibian skin fungal communities vary across host species and do not correlate with infection by a pathogenic fungus. Environ Microbiol 21(8):2905–2920. 10.1111/1462-2920.14682 31087743 10.1111/1462-2920.14682

[52] Albanese D, Fontana P, De Filippo C, Cavalieri D, Donati C (2015) MICCA: a complete and accurate software for taxonomic profiling of metagenomic data. Sci Rep 5(1):9743. 10.1038/srep09743 25988396 10.1038/srep09743 PMC4649890

[53] Fernandes AD, Macklaim JM, Linn TG, Reid G, Gloor GB (2013) ANOVA-like differential gene expression analysis of single-organism and meta-RNA-seq. PLoS ONE 8(7):e67019 23843979 10.1371/journal.pone.0067019 PMC3699591

[54] Metsalu T, Vilo J (2015) ClustVis: a web tool for visualizing clustering of multivariate data using principal component analysis and heatmap. Nucl Acids Res 43(W1):W566–W570. 10.1093/nar/gkv468 25969447 10.1093/nar/gkv468 PMC4489295

[55] Bates D, Mächler M, Bolker B, Walker S (2015) Fitting linear mixed-effects models using lme4. J Stat Softw 67(1):1–48. 10.18637/jss.v067.i01

[56] Wickham H, François R, Henry L, Müller K, Vaughan D (2023) dplyr: a grammar of data manipulation. R package version 1.1.4. https://dplyr.tidyverse.org. Accessed 8 Jan 2024

[57] Beule L, Karlovsky P (2020) Improved normalization of species count data in ecology by scaling with ranked subsampling (SRS): application to microbial communities. PeerJ 8:e9593. 10.7717/peerj.9593 32832266 10.7717/peerj.9593 PMC7409812

[58] Anderson MJ, Ellingsen KE, McArdle BH (2006) Multivariate dispersion as a measure of beta diversity. Ecol Lett 9(6):683–693. 10.1111/j.1461-0248.2006.00926.x 16706913 10.1111/j.1461-0248.2006.00926.x

[59] Sabino-Pinto J, Bletz MC, Islam MM et al (2016) Composition of the cutaneous bacterial community in Japanese amphibians: effects of captivity, host species, and body region. Microb Ecol 72:460–469. 10.1007/s00248-016-0797-6 27278778 10.1007/s00248-016-0797-6

[60] Belasen AM, Riolo MA, Bletz MC, Lyra ML, Felipe Toledo L, James TY (2021) Geography, host genetics, and cross-domain microbial networks structure the skin microbiota of fragmented Brazilian atlantic forest frog populations. Ecol Evol 11(14):9293–9307. 10.1002/ece3.7594 34306622 10.1002/ece3.7594 PMC8293785

[61] García-Sánchez JC, Arredondo-Centeno J, Segovia-Ramírez MG et al (2023) Factors influencing bacterial and fungal skin communities of montane salamanders of Central Mexico. Microb Ecol 86:670–686. 10.1007/s00248-022-02049-x 35705744 10.1007/s00248-022-02049-x

[62] Cruz-Cano R, Kolb M, Saldaña-Vázquez RA, Bretón-Deval L, Cruz-Cano N, Aldama-Cervantes A (2024) Existing evidence on the use of environmental DNA as an operational method for studying rivers: a systematic map and thematic synthesis. Environ Evid 13(1):2. 10.1186/s13750-024-00325-6 39294762 10.1186/s13750-024-00325-6 PMC11376102

[63] Dang TD, Searle CL, Blaustein AR (2017) Virulence variation among strains of the emerging infectious fungus *Batrachochytrium dendrobatidis* (Bd) in multiple amphibian host species. Dis Aquat Organ 124(3):233–239. 10.3354/dao03125 28492179 10.3354/dao03125

[64] Belasen AM, Russell ID, Zamudio KR, Bletz MC (2022) Endemic lineages of Batrachochytrium dendrobatidis are associated with reduced Chytridiomycosis-induced mortality in amphibians: evidence from a meta-analysis of experimental infection studies. Front Vet Sci 9:756686. 10.3389/fvets.2022.756686 35310410 10.3389/fvets.2022.756686 PMC8931402

[65] Spitzen-van der Sluijs A, Canessa S, Martel A, Pasmans F (2017) Fragile coexistence of a global chytrid pathogen with amphibian populations is mediated by environment and demography. P Roy Soc B-Biol Sci 284(1864):20171444. 10.1098/rspb.2017.1444 10.1098/rspb.2017.1444 PMC5647299 28978729

[66] Harrison XA, Price SJ, Hopkins K, Leung WT, Sergeant C, Garner TW (2019) Diversity-stability dynamics of the amphibian skin microbiome and susceptibility to a lethal viral pathogen. Front Microbiol 10:2883. 10.3389/fmicb.2019.02883 31956320 10.3389/fmicb.2019.02883 PMC6951417

[67] Bates KA, Clare FC, O’Hanlon S et al (2018) Amphibian chytridiomycosis outbreak dynamics are linked with host skin bacterial community structure. Nat Commun 9(1):693. 10.1038/s41467-018-02967-w 29449565 10.1038/s41467-018-02967-w PMC5814395

[68] Martínez-Ugalde E, Ávila-Akerberg V, González Martínez TM et al (2022) The skin microbiota of the axolotl *Ambystoma altamirani* is highly influenced by metamorphosis and seasonality but not by pathogen infection. Anim Microbiome 4(1):63. 10.1186/s42523-022-00215-7 36503640 10.1186/s42523-022-00215-7 PMC9743558

