# Supplementary material for: More Than Meets the Eye: Unraveling the Interactions Between Skin Microbiota and Habitat in an Opportunistic Amphibian
Source: Microb Ecol. 2025 Jan 24;87:176. doi: 10.1007/s00248-025-02489-1 (PMC11761533; doi:10.1007/s00248-025-02489-1)
Supplement: Supplementary file 1 [file 248_2025_2489_MOESM1_ESM.docx]

**Supplementary Information**

More than meets the eye: unraveling the interactions between skin microbiota and habitat in an opportunistic amphibian. Microbial Ecology. Zanovello Lucia, Galla Giulio, Girardi Matteo, Casari Stefano, Lo Presti Irene, Pedrini Paolo, Bertorelle Giorgio, Hauffe Heidi C.

Corresponding author information: Hauffe Heidi C., Conservation Genomics Research Unit and Animal, Environmental and Antique DNA Platform, Research and Innovation Centre, Fondazione Edmund Mach, San Michele all’Adige, TN, Italy; [heidi.hauffe@fmach.it](mailto:heidi.hauffe@fmach.it)

**Figures and figure captions**

**Fig.S1** Heatmap of the putative Bd-inhibitory ASVs (based on the public database built by Woodhams et al. [39]) found in both *B. variegata* skin samples and water samples. The upper rows show site description according to the four habitats of this study, codes of each site and detection of Bd. This plot was created with ClustVis and formatted using GIMP v2.10.18 (The GIMP Development Team. (2019). GIMP. Retrieved from <https://www.gimp.org>).

**Fig.S2** Venn diagrams representing microbial private and shared taxa across our samples. The upper diagrams represent (A) bacterial and (B) fungal taxa shared between sample types (B= *B. variegata* skin samples; W= water samples), for each habitat. The lower panels show (C) bacterial and (D) fungal taxa shared across habitats, considering only *B. variegata* skin samples (B_FLU= *B. variegata* skin samples from habitat FLU; B_VBE= from habitat VBE; B_AGR= from AGR; B_PAS= from PAS). These plots were created with the R package *ggplot2* and formatted using GIMP v2.10.18 (The GIMP Development Team. (2019). GIMP. Retrieved from <https://www.gimp.org>).

**Fig.S3** Alpha diversity estimates (PD: Faith’s phylogenetic diversity, Chao1 and Shannon) of *B. variegata* skin microbiota across four habitats (FLU, VBE, AGR, PAS) and for each sampled site in the Province of Trento, Italy. Upper panel: Bacteria; lower panel: Fungi. This plot was created with the R package *ggplot2* and formatted using GIMP v2.10.18 (The GIMP Development Team. (2019). GIMP. Retrieved from <https://www.gimp.org>).

**Fig.S4** Beta diversity estimates (left: Bray-Curtis; right: Jaccard) of *B. variegata* skin (dots) and water (triangles) samples across the four habitats (FLU, VBE, AGR, PAS). Upper panel: Bacteria; lower panel: Fungi. This plot was created with the R package ggplot2 and formatted using GIMP v2.10.18 (The GIMP Development Team. (2019). GIMP. Retrieved from https://www.gimp.org).
